# Racial Discrimination, Inflammation, Sleep, and Metabolic Syndrome From Adolescence to Young Adulthood

**DOI:** 10.1001/jamanetworkopen.2024.5258

**Published:** 2024-04-01

**Authors:** Ross H. McMillan, Celeste C. Thomas

**Affiliations:** Section of Gastroenterology, Hepatology & Nutrition, University of Chicago, Chicago, Illinois; Section of Endocrinology, Diabetes & Metabolism, University of Chicago, Chicago, Illinois

Although the term crisis may often be overused when describing the burden of disease processes, it is not an overstatement when describing the impact of metabolic syndrome (MetS) on the lives of US residents. One in 3 US residents is affected by MetS, “a cluster of metabolic dysregulations that includes insulin resistance, atherogenic dyslipidemia, central obesity and hypertension.”^1^ As such, it has been associated with a 2-fold increase in cardiovascular outcomes and 1.6-fold increase in all-cause mortality,^2^ not to mention its outsized economic impact.^3^ And while MetS affects people of all demographics, it is a constellation of metabolic factors that disproportionately affects individuals from racial and ethnic minority groups. Furthermore, while the effects of MetS may be most profound in adulthood, incidence of MetS in young adults is on the rise.^4^

Given the outsized impact of MetS on individuals who belong to minoritized racial and ethnic groups and the rise in incidence of MetS in younger adults, the authors of this study^5^ set out to explore the association between racial discrimination in one’s youth and MetS in early adulthood. By utilizing the Strong African American Families Healthy Adults (SHAPE) Project, a longitudinal cohort of Black adolescents, Heard-Garris et al^5^ were able to track the progression of MetS in a cohort of Black adolescents in the rural US South as they advanced into adulthood. They noted that more incidents of self-reported racial discrimination were associated with increased levels of systemic inflammation (measured by soluble urokinase plasminogen activator receptor [suPAR]) and poorer sleep (self-reported through a validated sleep scale), both of which were associated with a higher prevalence MetS. By associating discrimination with systemic inflammation and poor sleep during adolescence, and subsequently with MetS in early adulthood, the authors^5^ further explored the profound pathophysiological effects that racism and racial discrimination can have, especially early on in life.

Racial discrimination has long been one of several environmental factors associated with adverse health outcomes, particularly cardiometabolic syndromes, in racial and ethnic minority populations.^6^ Specifically, experiences with racism and racial discrimination have been linked to stress-related neurohormonal dysregulation, systemic inflammation, and allostatic overload, each of which serves as a proposed mechanism by which racial discrimination leads to adverse health outcomes.^6^ These effects can begin as early as childhood and can lead to objective findings such as increased inflammatory biomarkers like suPAR.^7^ Thus, racism and racial discrimination can act as drivers of a systemic inflammatory state, a known contributor to MetS.

In addition, prior research has identified disparities in sleep duration and sleep quality between racial and ethnic minority children and adolescents compared with White children.^8^ Given the association between poor sleep and MetS, improved sleep duration and sleep quality appear to be modifiable factors related to improved health outcomes, including MetS. Discrimination has also been linked to poor sleep and can act as a driver of sleep disparities.^9^

While not the first study to associate racial discrimination with disparities in sleep, systemic inflammation, or MetS, this study^5^ is unique in discussing their collective association and in doing so at such an early age. The imprint of racial discrimination and racism early in life can have profound implications on cardiometabolic health and mortality as patients age.
